# Endothelial Thioredoxin-Interacting Protein Depletion Reduces Hemorrhagic Transformation in Hyperglycemic Mice after Embolic Stroke and Thrombolytic Therapy

**DOI:** 10.3390/ph14100983

**Published:** 2021-09-27

**Authors:** Mohd. Salman, Saifudeen Ismael, Lexiao Li, Heba A. Ahmed, Michelle A. Puchowicz, Tauheed Ishrat

**Affiliations:** 1 Department of Anatomy and Neurobiology, The University of Tennessee Health Science Center, Memphis, TN 38163, USA; drsalmanmohd@gmail.com (M.S.); sismael@uthsc.edu (S.I.); lli84@uthsc.edu (L.L.); hahmed6@uthsc.edu (H.A.A.); 2 Department of Pediatrics, The University of Tennessee Health Science Center, Memphis, TN 38163, USA; mpuchowi@uthsc.edu; 3 Department of Pharmaceutical Sciences, The University of Tennessee Health Science Center, Memphis, TN 38163, USA; 4 Neuroscience Institute, University of Tennessee Health Science Center, Memphis, TN 38163, USA

**Keywords:** hyperglycemia, endothelial-specific TXNIP deletion, tissue plasminogen activator, hemorrhagic transformation, embolic stroke

## Abstract

We hypothesize that endothelial-specific thioredoxin-interacting protein knock-out (EC-TXNIP KO) mice will be more resistant to the neurovascular damage (hemorrhagic-transformation-HT) associated with hyperglycemia (HG) in embolic stroke. Adult-male EC-TXNIP KO and wild-type (WT) littermate mice were injected with-streptozotocin (40 mg/kg, i.p.) for five consecutive days to induce diabetes. Four-weeks after confirming HG, mice were subjected to embolic middle cerebral artery occlusion (eMCAO) followed by tissue plasminogen activator (tPA)-reperfusion (10 mg/kg at 3 h post-eMCAO). After the neurological assessment, animals were sacrificed at 24 h for neurovascular stroke outcomes. There were no differences in cerebrovascular anatomy between the strains. Infarct size, edema, and HT as indicated by hemoglobin (Hb)-the content was significantly higher in HG-WT mice, with or without tPA-reperfusion, compared to normoglycemic WT mice. Hyperglycemic EC-TXNIP KO mice treated with tPA tended to show lower Hb-content, edema, infarct area, and less hemorrhagic score compared to WT hyperglycemic mice. EC-TXNIP KO mice showed decreased expression of inflammatory mediators, apoptosis-associated proteins, and nitrotyrosine levels. Further, vascular endothelial growth factor-A and matrix-metalloproteinases (MMP-9/MMP-3), which degrade junction proteins and increase blood-brain-barrier permeability, were decreased in EC-TXNIP KO mice. Together, these findings suggest that vascular-TXNIP could be a novel therapeutic target for neurovascular damage after stroke.

## 1. Introduction

Diabetes is a rapidly rising threat that affects 30% of the over 8 million annual stroke patients in the United States [1]. Recent studies identified that hyperglycemia (HG), regardless of diabetes, significantly increases the risk and severity of acute stroke [2]. The American Diabetes Association reported that diabetic patients have a higher, two to four fold increased risk of ischemic stroke compared to those without diabetes and that increased blood glucose (BG) level is associated with high morbidity and mortality [3]. Several studies have reported that HG exacerbates the infarct size, hemorrhagic transformation (HT), and behavioral disabilities in rodents [4,5]. Consistently, worsened post-stroke outcomes and higher mortality were observed in HG patients [6]. This may occur because HG is associated with multiple disruptive mechanisms such as an increased level of glucocorticoids [7], increased anaerobic metabolism, increased focal ischemic stress response, hyperosmolarity, and lactic acidosis [2]. The clinical benefit of glucose normalization in the acute stroke setting is challenging, due to the increased risk of hypoglycemia [8]. Further, the limited understanding of the molecular mechanisms by which HG contributes to reperfusion injury is a critical barrier to progress in the development of new therapies for stroke.

Recombinant tissue plasminogen activator (tPA), the only FDA approved drug, remains the most effective treatment option for ischemic stroke. Unfortunately, tPA shows neurotoxic and pro-oxidant effects when it contacts endothelial cells and extracellular matrix, following effective recanalization in ischemic stroke [9,10]. It is also accepted that tPA further exacerbates neurovascular damage including edema, blood-brain barrier (BBB) dysfunction and HT and worsens functional outcomes in HG conditions [5]. Ischemia potentiates various deleterious processes, including vasogenic edema, poor cerebral blood flow, oxidative damage, and hemorrhagic conversion, all critical for BBB disruption [11,12]. The BBB integrity is dependent on cerebral microvessel endothelial cells containing tight junction (TJs) proteins such as claudin-5, occludin, and zonula occludens-1 (ZO-1), among others, and surrounded by pericytes and astrocytes [13]. It has also been demonstrated that ischemic stroke increases BBB permeability and significantly downregulates the TJ proteins, required for BBB integrity in rodent models [14]. Moreover, matrix metalloproteinases (MMPs) activation contributes to the degradation of the extracellular matrix around the blood vessels and have been implicated in post-ischemic BBB disruption. The underlying mechanism through which HG with tPA-reperfusion modulates these events after stroke is not fully understood. Hence, there is an urgent need to identify novel targets for potential therapies to mitigate these risks.

Our group has been leading studies that elucidated the pivotal role of neurovascular protection in stroke [15,16]. However, there is still a gap in our understanding of how inflammation and redox signaling contributes to neurovascular damage, particularly in HG conditions, to sustain neuronal injury and worsen stroke outcome. Thioredoxin-interacting protein (TXNIP) has emerged as a key pathological regulator of various diseases, associated with glucose and lipid abnormalities and inflammation, including diabetes mellitus and cerebrovascular diseases [17,18]. TXNIP is an endogenous inhibitor of the thioredoxin (TRX), that modulates the cellular redox signaling state and induces oxidative damage [19]. TRX is a thiol-oxidoreductase that plays a major role in cellular thiol-reduction, as part of an antioxidant defense system, and provides anti-inflammatory and anti-apoptotic effects at the cellular level [20]. TXNIP mediates pro-apoptotic and pro-inflammatory cascades in various stress-related diseases [21]. Taken together, TXNIP is a central signaling hub that links oxidative/glucose stress and inflammation to cellular injury, making it a “multiple pathways” target and thus a promising new approach for stroke therapy. Moreover, our most recent studies showed that acute HG involves TXNIP upregulation and exacerbates the inflammation and neurovascular damage associated with tPA-reperfusion in filament middle cerebral artery occlusion (MCAO) mouse model of ischemic stroke [5]. Previously, we and others demonstrated that stroke-induced TXNIP upregulation is associated with increased ischemic injury in normo-glycemic wild-type (WT) mice [20,22]. Similarly, global knocking down of TXNIP provides neuroprotection through inactivation of inflammasome-dependent and apoptotic inducing pathways in ischemic stroke [20]. We propose to extend this work to elucidate the involvement of vascular TXNIP in the neurovascular damage seen after hyperglycemic/diabetic stroke and tPA-reperfusion, using endothelial-specific TXNIP knock-out (EC-TXNIP KO) mice. In this study, we hypothesized that EC-TXNIP KO mice are more resistant to neurovascular damage associated with HG and tPA-reperfusion in a more clinically relevant murine model of embolic stroke.

## 2. Results

### 2.1. Post-Stroke Assessment of Weight Loss, Mortality Rate, and Neurological Deficits

**Figure 1:**
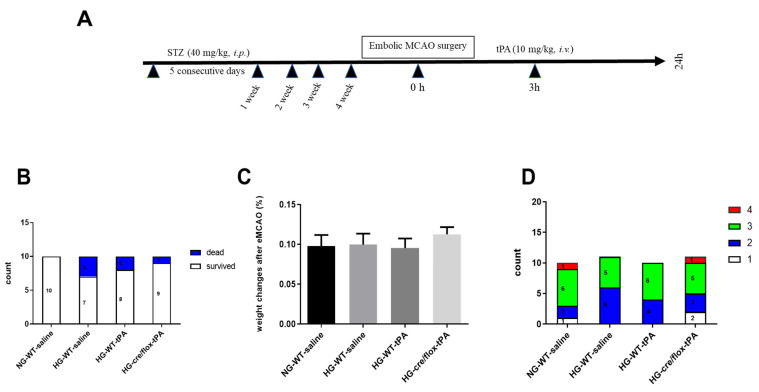
(**A**) Experimental timeline, (**B**) analysis of mortality rate, (**C**) weight loss and (**D**) neurobehavioral deficits after embolic stroke. Kruskal–Wallis test plus Dunn’s multiple comparisons; WT: wild-type; NG: normoglycemia; HG: hyperglycemia; tPA: tissue-type plasminogen activator; mean ± SEM; *n* = 7–10.

We determined the mortality rate; all the animals in the NG group survived while three animals died in the HG wild type non-tPA group, two animals in HG wild type with tPA group and one animal died in the EC-TXNIP KO (HG cre/flox-tPA) group. (Figure 1B). Additionally, we analyzed the weight loss and neurological deficit scores before animal’s sacrifice. There were no differences between any of the groups with respect to post-stroke weight loss (Figure 1C). Sensorimotor deficits, common in stroke patients, are a serious cause of functional disability and impaired quality of life. Therefore, we assessed neurological deficit post-stroke, using the Bederson’s scoring system, and no significant differences were noted between any of the groups (Figure 1D).

### 2.2. STZ-Based Hyperglycemic Profiling and Analysis of Glucose Variability upon Stroke with tPA-Reperfusion

**Figure 2:**
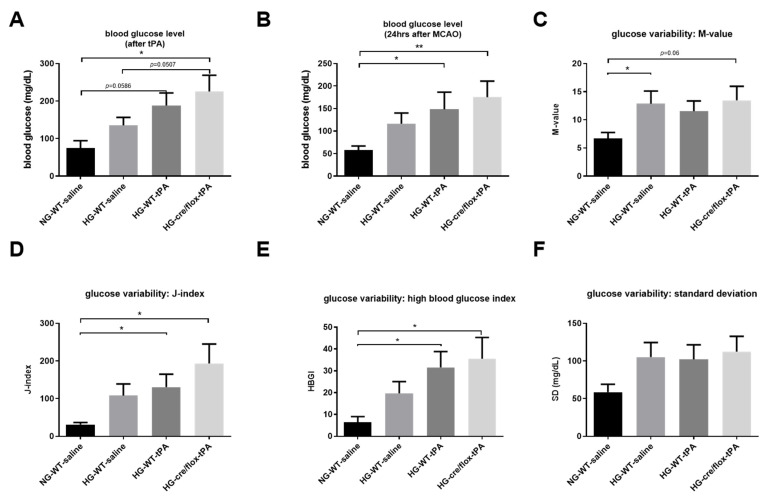
Analysis of STZ-based hyperglycemic profiling, and glucose variability. (**A**) EC-TXNIP KO HG mice have significantly higher glycemic level compared to WT HG after tPA-reperfusion (Kruskal–Wallis test plus Dunn’s multiple comparison, Mann–Whitney U test). (**B**) The tPA treatment may facilitate the maintenance of STZ-induced sub-acute hyperglycemic condition, and EC-TXNIP inhibition may exert a synergistic effect with tPA treatment in this regard (Kruskal–Wallis test plus Dunn’s multiple comparison). (**C**) The glucose variability was assessed via M-value. STZ-induced diabetic condition should undergo a higher glycemic variation in the first 24 h after stroke (Kruskal–Wallis test plus Dunn’s multiple comparison, Mann–Whitney U test). (**D**) The glucose variability was assessed via J-index. STZ-induced hyperglycemic condition should undergo a higher glycemic variation (Kruskal–Wallis test plus Dunn’s multiple comparison). The presence or absence of tPA treatment made no difference in this regard. (**E**) The glucose variability was assessed via standard deviation. STZ-induced hyperglycemic condition in EC-TXNIP KO mice should undergo a higher glycemic variation (Kruskal–Wallis test plus Dunn’s multiple comparison), whereas tPA treatment seems to be suppressive in this regard. (**F**) The prognosis of hyperglycemia was evaluated via high blood glucose index. STZ-induced hyperglycemia in EC-TXNIP KO as well as wild type mice treated with tPA is more likely to develop hyperglycemia when compared with non-STZ treated wild-type counterparts (Kruskal–Wallis test plus Dunn’s multiple comparison). WT: wild-type; NG: normoglycemia; HG: hyperglycemia; tPA: tissue-type plasminogen activator; mean ± SEM; * *p* < 0.05 & ** *p* < 0.01; *n* = 7–10.

The BG concentration was determined before the surgery, after tPA administration and right before sacrifice. EC-TXNIP KO mice showed a significantly (*p* < 0.05) higher BG level, after reperfusion with tPA, compared to NG and HG WT groups (Figure 2A). Moreover, BG level was significantly increased in the HG WT mice with tPA-reperfusion (*p* < 0.05) and EC-TXNIP KO mice (*p* < 0.01) compared to the NG group at 24 h post eMCAO (Figure 2B). We further analyzed the glucose variability over the entire study by calculating the M-value, standard deviation (SD), J-index, and high blood glucose index (HBGI). STZ treated animals experienced a much higher glycemic variability compared to the NG group, and this increased varia tion has been consistently indicated by the following metrics: M-value, J-index, SD, and HBGI (Figure 2C–F).

### 2.3. Effects of Sub-Acute HG on Infract Volume, Edema, and HT after eMCAO with tPA-Reperfusion

**Figure 3:**
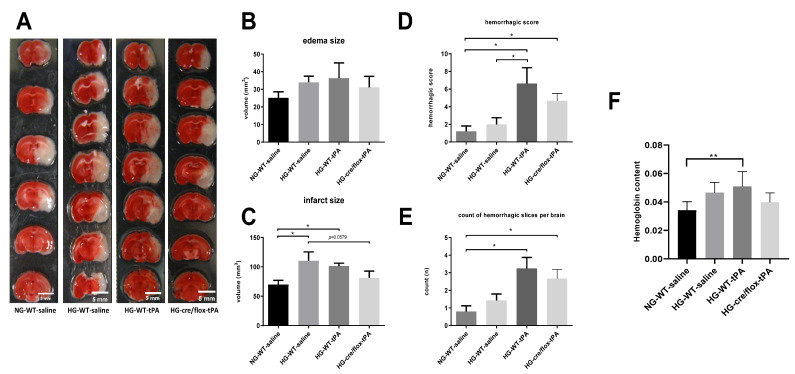
The impact of STZ-induced sub-acute HG in EC-TXNIP KO mice on edema formation, infarct size and HT after eMCAO and tPA-reperfusion. (**A**) The TTC-stained brain coronal slices were arrayed and digitized. Aligned images are the most representative for each group. (**B**) Higher level of brain edema was found in the HG group with tPA reperfusion than that NG and HG. However, EC-TXNIP KO mice showed a lower brain edema compared with the HG group with tPA-reperfusion. (**C**) As for the infarct size, the STZ-induced sub-acute HG leads to a significantly larger infarction. The deleterious effect due to HG, albeit insignificant, should not be disturbed by the absence of TXNIP in endothelial cells. (**D**) The qualitative hemorrhage scoring suggests that tPA treatment upon hyperglycemic condition led to more severe HT after stroke, and the absence of EC-TXNIP induces non-significant changes. (**E**,**F**) tPA-treatment following HG in EC-TXNIP KO mice exhibits a tendency of reduced ipsilateral hemoglobin content. When it comes to hemoglobin excess, the tendency no longer exists. Kruskal–Wallis test plus Dunn’s multiple comparisons; WT: wild-type; NG: normoglycemia; HG: hyperglycemia; HT: hemorrhagic transformation; tPA: tissue-type plasminogen activator; mean ± SEM; * *p* < 0.05 & ** *p* < 0.01; *n* = 7–9.

After cardiac perfusion, the brain coronal sections (1 mm thick) were immediately stained with 0.2% TTC solution at 37 °C. The stained brain slices containing white infarction (dead) and red (live) tissue were arrayed and digitized (Figure 3A). In comparison with NG mice, HG mice showed a larger edema resulting from the adverse effect of thrombolytic therapy. While EC-TXNIP KO mice showed a trend to decrease edema compared to HG with tPA-reperfusion (Figure 3B). Further, we calculated the volume of infarction in compliance with Swanson’s adjustment. Hyperglycemic conditions led to a significant increase in infarct size (*p* < 0.05), whilst post-MCAO reperfusion with tPA tends to prevent this enlargement of infarction. The therapeutic potential is of more significance as seen in EC-TXNIP KO mice (*p* = 0.05). While EC-TXNIP KO mice showed a trend to decrease in infarct volume in comparison with HG tPA-infused group (Figure 3C). Current data showed that post-stroke brain edema and infarct volume were exacerbated upon sub-acute hyperglycemic condition with tPA-reperfusion. While EC-TXNIP KO mice showed resistant from these events.

For hemorrhagic traces or patches, we used the same coronal brain TTC-stained slices. We observed that some slices showed the existence of hemorrhagic incidence after eMCAO that were susceptible to HG and/or tPA. The post-stroke hemorrhagic conversions and hemoglobin concentration were assessed in both qualitative and quantitative manners. Each brain section was subjected to the scoring system as reported by Kelly-Cobbs [23]. The sum of ipsilateral bleeding score and count of slices with hemorrhagic traces or patches for individual animal were performed. The statistical data was similar to the pattern seen in post-stroke edema formation and infarct volume (Figure 3D,E). Further, we analyzed the ipsilateral hemoglobin (Hb) content, which was significantly (*p* < 0.01) increased in HG with tPA compared to NG group. However, EC-TXNIP KO mice with tPA decreased the level of Hb content compared to HG with tPA group (Figure 3F).

### 2.4. Endothelial TXNIP Deletion Upregulates Junction Proteins Expression in HG Mice after eMCAO and tPA-Reperfusion

**Figure 4:**
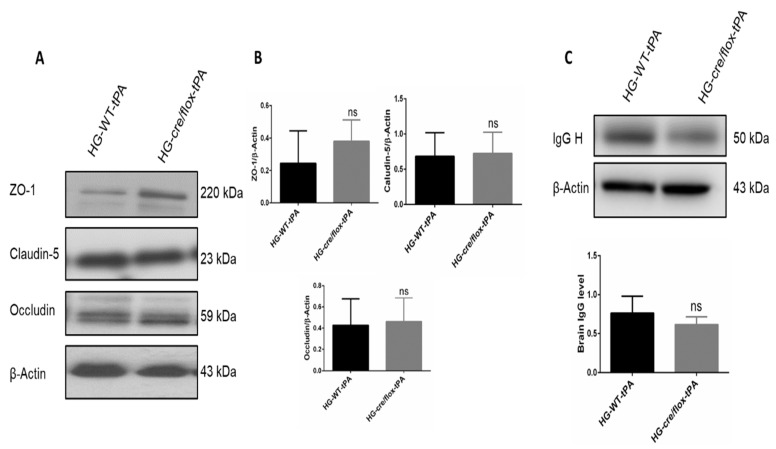
The impact of STZ-induced sub-acute HG in EC-TXNIP KO on TJs proteins and IgG expression after eMCAO and tPA-reperfusion. (**A**) Representative Western blot images of ZO-1, Claudin-5, and Occludin. (**B**) EC-TXNIP deletion non-significantly increased the expression of junctional proteins in post-stroke condition. (**C**) The expression level of IgG decreased in EC-TXNIP deletion mice when compared to the HG group mice with tPA reperfusion. The optical density of protein bands was analyzed and normalized to β-Actin (unpaired *t*-test). WT: wild-type; NG: normoglycemia; HG: hyperglycemia; tPA: tissue-type plasminogen activator; mean ± SD; ns = non-significant; *n* = 6.

To determine TJs protein expression (claudin-5, occludin, and ZO-1) Western blot analysis was performed on ipsilateral brain tissue at 24 h after eMCAO (Figure 4A). Alteration in TJs proteins lead to poor BBB function through alterations in permeability. EC-TXNIP KO mice showed higher expression of claudin-5 and occludin in STZ-based hyperglycemic mice compared to those treated with tPA. Similar regulatory pattern was seen in ZO-1 expression. STZ-based HG with tPA-reperfusion downregulated the expression of ZO-1 protein. While ZO-1 expression consistently increased in EC-TXNIP KO mice (Figure 4B). Further, we checked tissue IgG level, EC-TXNIP KO mice had a decreased IgG content compared to the HG with tPA group. Increased brain IgG level showed a greater degree of BBB damage (Figure 4C). Taken together, higher ipsilateral TJs protein expression and lower IgG content in EC-TXNIP deletion was associated with the comparative decline in BBB damage.

### 2.5. Endothelial TXNIP Deletion Downregulates the Expression of MMP-3/9 and VEGFA in HG Mice after eMCAO and tPA-Reperfusion

**Figure 5:**
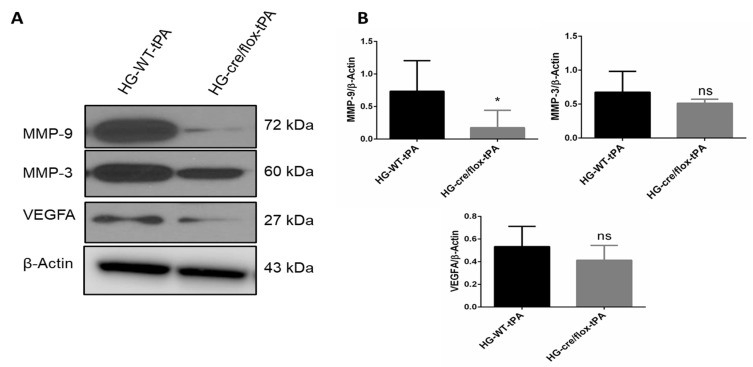
The impact of STZ-induced sub-acute HG in EC-TXNIP KO on MMP-9, MMP-3 and VEGFA expression after eMCAO and tPA-reperfusion. (**A**) Representative Western blot images of MMP-9, MMP-3 and VEGFA. (**B**) Bar graphs. EC-TXNIP deletion significantly decreased the expression of MMP-9 compared with HG group with tPA treatment. A non-significant downregulation was observed in MMP-3 and VEGFA proteins. The optical density of protein bands was analyzed and normalized to β-Actin (unpaired *t*-test). WT: wild-type; NG: normoglycemia; HG: hyperglycemia; tPA: tissue-type plasminogen activator; mean ± SD; ns = non-significant; * *p* < 0.05; *n* = 6.

The HG, stroke and thrombolysis are associated with endothelial dysfunction [8]. The expression of MMP-9 and MMP-3 in the ipsilateral hemispheres of HG mice were higher in WT hyperglycemic mice treated with tPA compared to WT normoglycemic controls. While EC-TXNIP KO mice showed a significant decrease (*p* < 0.05) in active MMP-9, and a trend to downregulate active MMP-3 expression compared to WT hyperglycemic mice treated with tPA. The expression of VEGFA was upregulated in HG with tPA-reperfusion, whilst EC-TXNIP KO showed lower VEGFA expression, as shown in Figure 5A,B.

### 2.6. Endothelial TXNIP Deletion Reduces Inflammation in HG after eMCAO and tPA-Reperfusion

**Figure 6:**
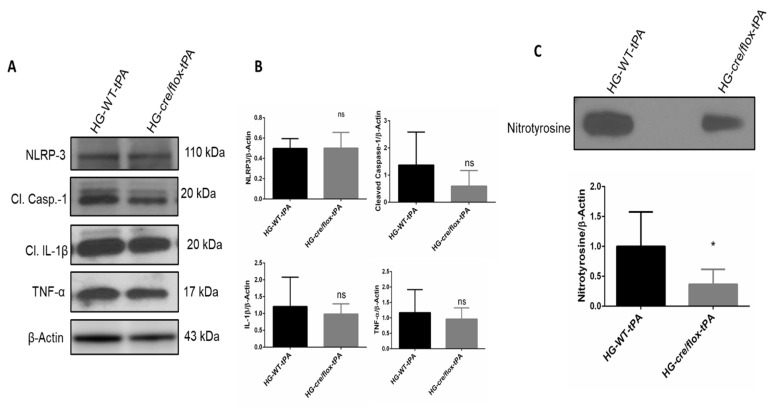
The impact of STZ-induced sub-acute HG in EC-TXNIP KO on inflammatory markers, cleaved-caspase-1 and nitrotyrosine after eMCAO and tPA-reperfusion. (**A**) Representative Western blot images of NLRP3, TNF-α, IL-1β, cleaved-caspase-1 and (**B**) bar graph. EC-TXNIP KO mice showed a downregulation in the TNF-α, IL-1β, cleaved-caspase-1proteins compared with the HG group with tPA treatment. While no changes were observed with respect to NLRP-3 protein compared with HG group with tPA treatment. (**C**) Representative image of immunoblot and bar graph of nitrotyrosine. EC-TXNIP KO mice showed a significantly downregulation nitrotyrosine level compared with HG group with tPA treatment. The optical density of protein bands was analyzed and normalized to β-actin (unpaired *t*-test). WT: wild-type; NG: normoglycemia; HG: hyperglycemia; tPA: tissue-type plasminogen activator; mean ± SD; ns = non-significant; * *p* < 0.05; *n* = 6.

We further examined the inflammatory pathway mainly involving NLRP3 inflammasome and pro-inflammatory cytokines through detecting protein expression of NLRP3, TNF-α, IL-1β and cleaved-caspase-1 in the ipsilateral hemisphere. There was no difference observed in the expression level of NLRP3 in HG mice and EC-TXNIP KO with tPA-reperfusion. However, the expression level of TNF-α and IL-1β was downregulated in EC-TXNIP KO mice compared to HG with-tPA reperfusion. Similar to the pattern of TNF-α and IL-1β, EC-TXNIP KO mice tends to downregulate cleaved-caspase-1 expression compared to HG with tPA-reperfusion. Taken together, EC-TXNIP deletion attenuates post-stroke inflammation during HG with tPA treatment, as shown in Figure 6A,B.

### 2.7. Endothelial TXNIP Deletion Attenuates Nitrotyrosine Formation in Brains of HG Mice, after eMCAO and tPA-Reperfusion

Next, we evaluated the formation of nitrotyrosine at 24 h after eMCAO. Nitrotyrosine is a product of tyrosine nitration, a marker of oxidative damage and inflammation. The level of nitrotyrosine was significantly (*p* < 0.05) lower in EC-TXNIP KO mice with tPA-reperfusion compared with WT HG mice with tPA-reperfusion (Figure 6C).

### 2.8. Endothelial TXNIP Deletion Downregulates the Protein Expression Levels of Cleaved-caspase-3, and Cleaved PARP-1 in Brains of HG Mice, after eMCAO and tPA-Reperfusion

**Figure 7:**
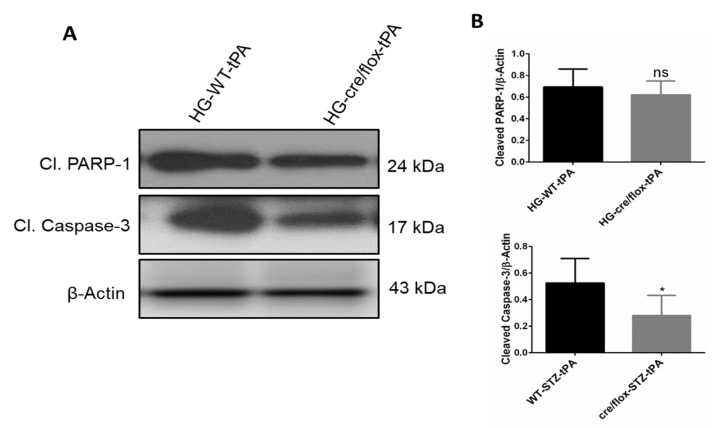
The impact of STZ-induced sub-acute HG in EC-TXNIP KO on cleaved PARP-1 and caspase-3 after eMCAO and tPA-reperfusion. (**A**) Representative Western blot images of cleaved PARP-1, cleaved-caspase-3 and (**B**) bar graphs. EC-TXNIP mice showed a significant downregulation of cleaved-caspase-3 level compared to HG plus tPA treatment group. While cleaved PAPR-1 was non significantly down regulated compared to HG group with tPA. The optical density of protein bands was analyzed and normalized to β-Actin (unpaired t test). WT: wild-type; NG: normoglycemia; HG: hyperglycemia; tPA: tissue-type plasminogen activator; mean ± SD; ns = nonsignificant * *p* < 0.05; *n* = 6.

We next detected the protein expression of apoptotic markers cleaved-caspase-3 and cleaved PARP-1 in the ipsilateral hemispheres. Increased expression of these proteins is associated with high rate of apoptosis. EC-TXNIP KO HG mice showed a significant downregulation (*p* < 0.05) in cleaved-caspase-3, after tPA reperfusion, compared to the WT HG mice treated with tPA. However, no significant changes were observed in cleaved PARP-1 expression between the groups (Figure 7A,B). Taken together, HG mice with EC-TXNIP deletion appeared to have a lower rate of apoptosis compared to WT HG mice treated with tPA.

## 3. Discussion

**Figure 8:**
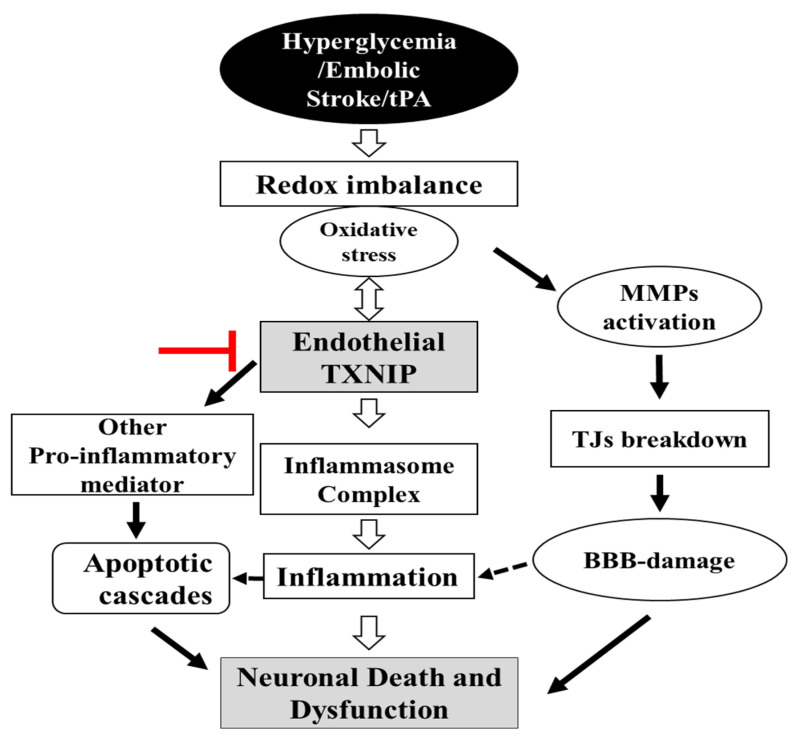
Schematic diagram illustrating postulated mechanism by which EC-TXNIP KO provides protection against HG with tPA reperfusion following embolic stroke: A variety of different activated pathways including inflammation, apoptotic cascades and MMPs activation, TJs breakdown, and BBB-damage all belong to the HG, which further trigger followed by tPA-reperfusion. However, EC-TXNIP deletion attenuates neuronal cell death and dysfunction through the inhibition of these pathways following HG with tPA-reperfusion in embolic stroke model in mice. BBB: blood-brain barrier; HG: hyperglycemic; MMPs: matrix metalloproteinases TXNIP: thioredoxin-interacting protein; tPA: tissue plasminogen activator.

In the current study, we demonstrated that mice with an endothelial cell selective deletion of TXNIP are more resistant to the neurovascular damage associated with STZ-based HG with tPA-reperfusion in a murine model of embolic stroke. Recent studies demonstrated that post-stroke HG was associated with coagulation dysfunction, cerebrovascular inflammation, HT, and poor BBB function that significantly increases the risk and severity of acute stroke [5,24,25]. Although, tPA thrombolytic therapy is commonly used in stroke therapy, it further exacerbates these events during HG conditions [5,26,27]. Our data show that EC-TXNIP KO ameliorated HT and IgG extravasation by improving BBB integrity (TJ proteins) and inhibiting neuroinflammation in STZ-based sub-acute HG with tPA-reperfusion following embolic stroke. We also found that EC-TXNIP deletion reduces the expression of MMP-3/9 and VEGFA proteins. Activation of these proteins promote BBB disruption, infarct size, edema and HT in the acute phase of stroke [28,29]. Together, our data suggest that EC-TXNIP deletion facilitates resistance against the HT and neurovascular damage associated with hyperglycemic conditions with tPA-reperfusion following embolic stroke (Figure 8).

In past decades, most studies have focused on the effects of hyperglycemia per se on ischemic stroke pathogenesis but not with thrombolytic therapy. However, the molecular mechanism underlying hyperglycemic condition with tPA-reperfusion is poorly understood. We reported for the first time that HG with tPA-reperfusion increased the expression level of TXNIP protein, which in turn increased, oxidative damage, inflammation and neuronal cell death and dysfunction in ischemic stroke [5]. The mechanism(s) of action by which TXNIP modulates neuronal damage in HG, which is further exacerbated with thrombolytic therapy after embolic stroke are not completely understood. Following oxidative stress, TXNIP is involved in the different signaling pathways which are engaged in a diversity of neuronal dysfunction [20,30].Moreover, TXNIP is required for activation of the NOD-like receptor protein 3 (NLRP3)-inflammasome, a multi-protein complex involved in instigating inflammation and immune regulation in several neurovascular injury models including stroke [5,20,21,31]. Activation of TXNIP/NLRP3 inflammasome convert pro-caspase-1 into cleaved-caspase-1 and inhibits the TRX resulting oxidative stress in acute HG suture stroke model [5]. Cleaved-caspase-1 further cleaves pro-IL-1β into the active pro-inflammatory cytokine, mature IL-1β, which is then released into the extracellular space and plays an important role in inflammation and neuronal apoptosis [20]. Moreover, preclinical studies have shown that TXNIP also increases the expression level of TNF-α in hyperglycemic condition with tPA-reperfusion in stroke [5,32]. Our results demonstrate that expression level of cleaved-caspase-1, IL-1β and TNF-α were decreased in HG EC-TXNIP KO mice with tPA-reperfusion, despite no change in NLRP-3 expression.

Chronic hyperglycemia has long been associated with brain endothelial complications in diabetes and metabolic disorders through different signaling cascades, including the TXNIP/NLRP-3 pathway [5,18]. Several studies have implicated the involvement of HG in neurovascular damage following ischemic stroke, HG further increased the oxidative stress, inflammatory responses as well as increased the BBB permeability [33]. This may lead to a rapid activation of MMPs and disruption in TJs proteins after cerebral ischemia. TJs proteins are important structural elements of BBB, which seal the gaps between adjacent endothelial cells and thus maintain paracellular integrity [34]. Down regulation of TJs indicate poor BBB function [35]. Our results demonstrate that EC-TXNIP deletion restored the loss of these proteins in hyperglycemic condition with tPA-reperfusion following embolic stroke. Further, activated MMPs could directly damage neurovascular components including junctional proteins and involved in hemorrhage following ischemic stroke [36,37]. Moreover, evidence suggested that tPA-reperfusion upregulates various members of the MMPs specially MMP-2 and MMP-3 [15]. Vascular endothelial growth factor (VEGF) is a potent inducer of vascular permeability and plays a critical role in causing BBB disruption and cerebral edema [38]. Experimental evidence has shown that VEGF down-regulates the expression of TJs proteins in brain microvasculature [39]. In this study, we observed that MMP-3/9 and VEGFA expression were downregulated in EC-TXNIP KO mice with HG and tPA-reperfusion, which correlates with improved BBB function after embolic stroke. Our results support the concept that tPA-reperfusion in HG mice promotes HT and BBB damage after embolic stroke, possibly through TXNIP and upregulating MMP-3/9, and VEGFA expression. To further calculate the involvement of TXNIP in HT and BBB disruption following hyperglycemic condition with tPA-reperfusion, we utilized an EC-TXNIP KO mouse model. We found that inhibition of TXNIP attenuated HT, BBB dysfunction and brain infarction in hyperglycemic condition with tPA-reperfusion following embolic stroke, indicating that endothelial TXNIP inhibition could be an important target for neurovascular protection in embolic stroke.

Ischemic stroke contributes to neuronal loss via different mechanisms especially apoptosis in the penumbral region of the brain [40]. Physiologically, apoptosis is essential for proper homeostasis and survival in different multi-organisms [41], however, uncontrolled apoptosis results in increased damage often leading to the worse outcomes in post-stroke [42]. There are two major pathways: caspase-dependent and caspase-independent. Few studies reported the influence of HG on apoptosis in brain endothelial cells; however, hyperglycemic condition-induced endothelial apoptosis has been demonstrated in different cell types [43,44]. In the current study, we examined the expression of cleaved-caspase-3 and cleaved PARP-1 protein. We found that HG with tPA-reperfusion had higher expression level of these proteins comparatively EC-TXNIP KO mice which indicates that chronic HG resulted in increased apoptotic cell death. However, endothelial TXNIP deletion significantly reduced the expression of cleaved-caspase-3 and slightly reduced the expression of cleaved PARP-1. These results suggest that HG with tPA treatment induces apoptotic cell death through the activation of a caspase-dependent pathway in embolic stroke. On the other hand, the effect of HG with tPA treatment on caspase-independent pathway should be investigated.

## 4. Materials and Methods

### 4.1. The Animals and Study Design

Wild-type male C57Bl/6 (Jackson Laboratory, Bar Harbor, ME, USA) mice aged 8–12 weeks were used in the current study. For EC-TXNIP KO, *txnip* Flox mice (Jackson Lab: 016847) were crossed with endothelial specific cre (Cdh5 Cre) mice (Jackson Lab: 006137) to generate EC-TXNIP KO and confirmed by standard PCR using genomic DNA isolated from tail snips. All experimental and surgical procedures were conducted in compliance with the regulations of the Institutional Animal Care and Use Committee (IACUC) at UTHSC and the ARRIVE (Animal Research: Reporting in Vivo Experiments) guidelines. Studies followed the highest standards and experimental rigor STAIR criteria (NIH guide for care and use of laboratory animals, anesthetized and temperature controlled, housing and husbandry, randomization, blinding, interpretation and statistical analysis). The animals were housed in standard humidity (45–50%) and temperature (21–25 °C) and 12 h photo cycle with food and filtered-water ad libitum. The animals were assigned into four different experimental groups including normoglycemic-wild type control (NG-WT), HG-wild type (HG-WT), HG-wild type animals treated with tPA (HG-WT-tPA), and HG-EC-TXNIP KO animals treated with tPA (HG-cre/flox-tPA). Sub-acute HG was created by intraperitoneally (IP) injected streptozotocin (STZ; 40 mg/kg in 0.1 M citrate buffer), administered for five consecutive days. Blood glucose was measured from the tail prick using a glucometer (Contour Blood Glucose Monitoring System). Four weeks after confirming HG, mice were subjected to embolic middle cerebral artery occlusion (eMCAO), followed by reperfusion with tPA (10 mg/kg, in sterilized water, Alteplase, NDC 50242-044-06, Genentech, CA, USA) at 3 h after eMCAO. Post-stroke neurological outcomes were evaluated before the mice were sacrificed at 24 h after eMCAO (Figure 1A).

### 4.2. Induction of Focal Embolic Stroke Model

Preparation of blood clots: Blood clots were prepared following the method of Ren et al. with some minor modifications [45]. In brief, the experimental animals were anesthetized with 1.5% isoflurane. Fresh arterial blood was harvested from the cardiac ventricle through a 1 mL syringe with 21-gauge needle. An amount of 200 μL fresh arterial blood was mixed with 10 μL fibrinogen (#341576, Millipore, Billerica, MA, USA) in a 0.5 mL micro-centrifuge tubes, and immediately injected into the PE-50 catheter. The catheter filled with blood was placed at room temperature overnight and then stored at 4 °C. The clot was ready for MCAO surgery after being washed in normal phosphate buffer saline (PBS), stained with 1% Evans Blue solution in normal saline (#E2129, Sigma-Aldrich, Burlington, MA, USA), and loaded into the catheter in 0.9% saline. The length of clot inside the polyethylene catheter was measured prior to clot delivery. The baseline clot size was defined as the product of its length multiplied by the internal diameter of the catheter. The criterion for successful delivery of the clot was for it to be partially visible inside the middle cerebral artery and/or circle of Willis after the mouse brain was harvested. The rate of clot ablation over 24 h after stroke was assessed.

eMCAO surgery: All experimental animals were anesthetized using 2–3% isoflurane inhalation with the help of a nose cone. A mid-line incision was made on the neck. Subcutaneous soft tissues were subjected to blunt separations. The right common carotid artery (CCA) was carefully separated from its adjacent vagal nerve, which was carefully protected from surgical damage. The occipital artery was ablated before the bifurcation of internal carotid artery (ICA) distal part and the pterygopalatine artery (PPA) was visible. Both the CCA and ICA were clipped, and a cut was prepared along the ECA residue for the insertion of catheter (#BB31695-PE/08, Scientific Commodities, Lake Havasu City, Arizona, US). The eMCAO was achieved via the delivery of 3–4 mm long clot through the catheter into the internal carotid artery to block the origin of the middle cerebral artery. After the surgery, animals were kept under infrared light for their comfort and recovery from anesthesia. The decline in regional cerebral blood flow was detected using a multichannel laser Doppler flowmetry (Periflux 5000 Master, Perimed AB, Stockholm, Sweden) equipped with a probe (PROBE 418-1 Master Probe, Perimed AB, Stockholm, Sweden).

Surgical exclusion criteria: For consistent eMCAO, only animals with a Bederson score of ≥ 2 at 24 h after eMCAO were included in further analysis. For the tPA group, only animals with a score of 3 prior to reperfusion with tPA were included for further analysis. Mice that did not develop sufficient deficits, died during the observation period and developed a subarachnoid hemorrhage after embolization were excluded from the study.

### 4.3. Neurobehavioral Assessment

Neurological deficits were evaluated in a blinded manner at 24 h after eMCAO. According to the modified neurological deficit of Bederson scoring system, [46] animals with no apparent deficits obtained, “0”; signs of forelimb flexion, “1”; reduced resistance to lateral push, “2”; circling, “3”.

### 4.4. Assessment of Infarct Size and Edema

At 24 h after eMCAO, the animals were deeply anesthetized with ketamine/xylazine mixture (85% and 15%, respectively) and transcardially perfused with ice cold PBS. Animals were then decapitated, and the brain tissues were isolated carefully. The 1-mm thick coronal sections from each brain were stained with 0.2% 2,3,5-triphenyltetrazolium chloride solution (TTC, Sigma-Aldrich, St. Louis, MO, USA) for 20 min at room temperature. Infarction and the entire hemisphere volumes were blindly measured using ImageJ software. The calculation of global infarct volume followed Cavalieri’s principle in the three-dimensional case (or “trapezoidal rule”). The infarct volume was adjusted for edema formation in compliance with Swanson’s correction [47].

### 4.5. Evaluation of Hemoglobin Content

Cerebrovascular disruption and red blood cell extravasation into the brain parenchyma was quantified using a colorimetric hemoglobin detection assay (QuantiChrom Hemoglobin Assay Kit, BioAssay Systems; Haywood, CA, USA) following manufacturer’s instructions to assess post-stroke hemorrhagic conversions. The samples were subjected to the colorimetric reactions yielding a uniformly colored hemoglobin and read at 562 nm using a standard microplate reader (Synergy HT, BioTek instruments). Hemoglobin concentration was recorded in μg/dL, based on a standard sample and the result was represented as hemoglobin content. Alternatively, each TTC-stained section was evaluated for hemorrhage score, according to the previous definition as follows [48]; 0 = normal ischemic damage—no hemorrhage; 1 = dispersed individual petechiae; 2 = confluent petechiae; 3 = hemorrhagic infarction; 4 = large cerebral hemorrhage; 5 = animal found dead due to intra cerebral hemorrhagic before planned termination; 5.5 = hemorrhage to non-ischemic brain tissue.

### 4.6. Slot Blot for Nitrotyrosine

Nitrotyrosine (NT) immunoreactivity was measured by slot blot analysis. In brief, brain tissue homogenate was prepared in lysis buffer and 25 μg protein were immobilized onto a nitrocellulose membrane from each group using a slot blot micro-filtration unit. After blocking with 5% non-fat milk, the membrane was incubated with a primary antibody against nitrotyrosine (05-233; Millipore, Saint Louis, MO, USA) followed by HRP conjugated secondary antibody. Bands were visualized by enhanced chemiluminescent substrate system (Thermo Fisher scientific). The optical density was quantified using ImageJ software.

### 4.7. Western Blot Analysis

For Western blotting analyses, peri-infarct (penumbra) cortical regions presenting in coronal slices (approximately from 0.5 mm to -3.5 mm bregma distance) were homogenized in RIPA buffer as previously described [5]. Twenty-micrograms of proteins were loaded into each well and separated by SDS-PAGE and transferred to PVDF membranes. The membranes were blocked for non-specific binding and incubated with primary antibodies against TXNIP (1:1000, NBP1-54578, Novus Biologicals) NLRP3, cleaved-caspase-1 (1:1000; AG-20B-0014, AG-20B-0042, Adipogen life sciences, San Diego, CA, USA), IL-1β (1:1000, #12242 Cell signaling technology, Danvers, MA, USA), claudin-5 (1:1000, #4C3C2, Thermo Fisher Scientific), occludin (1:1000, #OC-3F10, Thermo Fisher Scientific, Waltham, MA, USA), ZO-1 (1:1000, #40-2200, Thermo Fisher Scientific), VEGFA (1:1000, AB1876-I, Millipore), MMP-9 (1:1000, #PA5-13199, Thermo Fisher Scientific, Waltham, MA, USA), MMP-3 (1:1000, NBP2-75931, Novus Bilogicals), cleaved PARP-1 (1:1000, ab32064, Abcam, Cambridge, UK), TNFα (1:1000; Cell Signaling Technology, Danvers, MA, USA), and β-actin (1:1000, #A5316 Sigma) at 4 °C overnight. The membranes were washed in TBS-T and incubated with horseradish peroxidase-conjugated secondary antibodies (1:10,000, Sigma-Aldrich, Burlington, MA, USA). The bands were then visualized by means of an enhanced chemiluminescent substrate system (Thermo Fisher scientific, Waltham, MA, USA). Protein expression levels were analyzed densitometrically, using ImageJ software. The data were normalized with loading controls.

### 4.8. Statistical Analysis

The current study involves normal distribution test, unpaired *t*-test, Mann–Whitney U test. All these analyses were facilitated by the statistical software GraphPad Prism 6 that also aided the data visualization in this study. In addition, the Grubb’s test was employed to determine outliers. The results were expressed as mean ± SEM and SD. Significance was defined by *p* < 0.05.

## 5. Conclusions

In conclusion, we found that HG with tPA-reperfusion significantly increases brain infarct size, BBB disruption and HT in the brain after embolic stroke. The inhibition of vascular TXNIP might represent a novel strategy for reducing ischemic damage, particularly BBB disruption and HT, during HG conditions with tPA-reperfusion. Our study indicates that targeting EC-TXNIP may provide a novel neurotherapeutic option for preventing HT in ischemic stroke patients with HG.

## Data Availability

Data is contained within the article.
